# Drivers for change in primary care of diabetes following a protected learning time educational event: interview study of practitioners

**DOI:** 10.1186/1472-6920-8-4

**Published:** 2008-01-19

**Authors:** Aloysius Niroshan Siriwardena, Jo B Middlemass, Kate Ward, Carol Wilkinson

**Affiliations:** 1 School of Health and Social Care, University of Lincoln, Lincoln, UK; 2 Nottingham Primary Care Research Partnership, Nottingham County Teaching Primary Care Trust, Nottingham, UK; 3 Research, Lincolnshire Teaching Primary Care Trust, Lincoln, UK; 4 Independent researcher, Lincoln, UK

## Abstract

**Background:**

A number of protected learning time schemes have been set up in primary care across the United Kingdom but there has been little published evidence of their impact on processes of care. We undertook a qualitative study to investigate the perceptions of practitioners involved in a specific educational intervention in diabetes as part of a protected learning time scheme for primary health care teams, relating to changing processes of diabetes care in general practice.

**Methods:**

We undertook semistructured interviews of key informants from a sample of practices stratified according to the extent they had changed behaviour in prescribing of ramipril and diabetes care more generally, following a specific educational intervention in Lincolnshire, United Kingdom. Interviews sought information on facilitators and barriers to change in organisational behaviour for the care of diabetes.

**Results:**

An interprofessional protected learning time scheme event was perceived by some but not all participants as bringing about changes in processes for diabetes care. Participants cited examples of change introduced partly as a result of the educational session. This included using ACE inhibitors as first line for patients with diabetes who developed hypertension, increased use of aspirin, switching patients to glitazones, and conversion to insulin either directly or by referral to secondary care. Other reported factors for change, unrelated to the educational intervention, included financially driven performance targets, research evidence and national guidance. Facilitators for change linked to the educational session were peer support and teamworking supported by audit and comparative feedback.

**Conclusion:**

This study has shown how a protected learning time scheme, using interprofessional learning, local opinion leaders and early implementers as change agents may have influenced changes in systems of diabetes care in selected practices but also how other confounding factors played an important part in changes that occurred in practice.

## Background

A number of protected learning time (PLT) schemes in primary care have been set up across the United Kingdom. Published evaluations have focused on organisational aspects and the views of participants of the benefits, problems and possible effects of such schemes [1-6]. There has been little published evidence of their impact on processes of care or improved patient outcomes. Reported effects on prescribing behaviour or process changes have not adequately accounted for secular (underlying) trends in performance.

It has been argued that studies of educational interventions should evaluate change in a geographical area (rather than a single practice) targeting an identifiable learning need which if addressed could lead to real improvements in patient outcomes [7]. In the case of interprofessional learning, "when [members or students of] two or more professions learn with, from and about one another to improve collaboration and the quality of care [8]" it should also be focused on a relevant problem appropriate for the multiprofessional group [4]. In order to do this a mixed methods study was conducted into the effect on practice prescribing and behaviour of an educational session on diabetes care provided by Lincolnshire TARGET (Time for Audit, Review, Guidelines, Education and Training), set up as an multidisciplinary protected learning time (PLT) scheme, and innovative in that it involved all general practices with their associated primary care teams in a large rural county of the East Midlands, United Kingdom.

The educational session was centred around the HOPE study which provided evidence that patients with coexisting diabetes and hypertension or other cardiovascular risk factors should be treated with an ACE inhibitor at a therapeutic dose (specifically ramipril 10 mg) [9] to reduce cardiovascular morbidity and mortality. An interrupted time series analysis showed a significant increase in ACE inhibitor prescribing across the county, taking into account secular change, following the educational intervention (odds ratio 1.50, 95% CI 1.07–1.93) with an increase in prescribing of ramipril by 52,345 items (31,132 items at 10 mg) at a cost of £292 k to £460 k depending on formulation [10].

The aim of this parallel qualitative study was to investigate the perceptions of practitioners on the effect of the educational event relating to their processes of diabetes care. We were interested in what practices did, if anything, to implement and sustain change as a result of the educational intervention, what the barriers to change were, what other external factors may have led to change, and which elements of the educational intervention were helpful or not helpful.

## Methods

### Lincolnshire TARGET

Lincolnshire TARGET was set up in 2000 with the aim of providing needs based learning for general practitioners, community nurses and administrative staff working in primary care during working hours. This was achieved by using local out-of-hours cooperatives and other internal arrangements in group practices to provide primary care services during educational sessions. Each session was organised and delivered by a team of educators, led by a clinical director with administrative support, and focused on a topic based on both local practitioner need and national priorities. The sessions involved a combination of lectures delivered by local, regional or national opinion leaders and facilitated small interdisciplinary group work involving medical, nursing and administrative staff. At the end of each session individual primary care teams worked in small groups to discuss how they might implement change as a result of the education. TARGET included educational sponsorship from a number of pharmaceutical companies and promotional stands were also a feature of the sessions. Lunch was provided before the educational meeting began but no other incentives were offered to encourage attendance.

### Intervention of interest

The educational session on diabetes was delivered in November 2001 and lasted about 2.5 hours. It consisted of a welcome session outlining the objectives for the afternoon, an opening talk to set the scene of diabetes in general practice followed by parallel talks for clinicians and administrative staff. The first session for clinicians was designed to look at diabetes prevalence and screening following which a local general practitioner who had successfully implemented the findings from HOPE into his own practice was able to describe practical steps leading to improvements in ACE inhibitor prescribing for diabetes within his practice.

For other (including nonclinical) staff a talk was given from the patient's perspective by a speaker from Diabetes UK, a talk on the diabetic clinic was given by a local specialist diabetic nurse and a practical session looking at computer data, risk assessment and routine procedures, for example in reception, was given. Finally practices teams met together as a group, to discuss the barriers to improving practice processes for diabetes care, including implementation of HOPE, and considered an action plan of how they might overcome barriers and implement better systems of care. Each plan was fed back to the whole audience.

### Selection and interviews

Interviewees were chosen purposively from selected practices. Nurses and general practitioners providing diabetes care in practices were specifically chosen because they were more likely to have in-depth knowledge of changes in processes of care whether related to the intervention or not. Practices were selected according to the extent of change in prescribing following the educational intervention. Stratified sampling was used to get a variety of perspectives because the quantitative study showed that there were differences in change in prescribing rates in practices in temporal relation to the intervention. Practices were therefore divided into four strata depending on change in prescribing as follows [10]:

Stratum 1 (S1): little increase in prescribing before or after the educational intervention

Stratum 2 (S2): some increase in prescribing before and after

Stratum 3 (S3): little change before but a great deal after

Stratum 4 (S4): a great deal of change before and after

One of the authors (KS) selected three practices, ordered at random, from each of the four strata and invited them to take part in the qualitative interview study. If a practice declined to take part the next practice on the list from that stratum was invited. A total of twelve interviews were carried out, three from each of the four strata. Interviewees (practices) and interviewers were blinded to the practice stratum so that neither the practice, nor the interviewers were aware of the stratum from which the practice was drawn at the time of the interview (see Table 1). The audiotape from one practice in stratum 2 was inadvertently damaged precluding analysis. Informed consent was sought from participants.

**Table 1:** Characteristics of participating practices

**Stratum**	**Stratum 1**: little increase before or after (n = 50; non consenting 2)	**Stratum 2**: some increase before or after (n = 27; non consenting 23)	**Stratum 3**: little increase before, lots after (n = 12, non consenting 4)	**Stratum 4**: great increase before and after (n = 14, non consenting 5)
**Interviewed practices **(n = 11)	3	2	3	3
Characteristics: of interviewees and interviewed practices	a. GP, semirural, 5 partner ELPCT	a. GP, urban, 3 partner, WLPCT	a. Nurse, semirural, 4 partner WLPCT	a. GP, semirural, 9 partner training WLPCT
	b. GP, semirural, single handed ELPCT	b. GP, rural 4 partner ELPCT	b. Nurse, rural, 6 partner ELPCT	b. GP, semirural, 4 partner ELPCT
	c. GP, urban, 3 partner WLPCT		c. GP, urban, 8 partner, training, LSWPCT	c. GP, rural, 6 partner ELPCT
**All practices **(n = 103)				
Single handed (n = 20)	16	3	0	0
Dispensing (n = 59)	26	12	9	12
Training (n = 15)	4	6	2	3
At least one GP attending (n = 68)	25	24	8	11
At least one PN/HV attending	28	20	8	8

### Interviews and data analysis

The interviews looked for perceptions of change in diabetes care and facilitators of change. This included not only the educational session but also any other factors that may have prompted change, barriers to change and evidence of practice interventions to implement change (Table 2). One-to-one in depth interviews took place at the practice premises in 2003, within 18 months of the educational session. They were 45 to 90 minutes in duration and conducted individually by two researchers. They were tape recorded and transcribed in full. Qualitative data from the transcripts were analysed using specific software (QSR N6). A sample of the transcripts was independently examined by all members of the project team and categories derived by induction. Categories were decided and grouped into themes through discussion. Themes were identified in the context of the stratum and the professional discipline of the interviewee and agreed through examination of transcripts by all members of the team.

**Table 2:** Semistructured interview schedule

1. What have been the major changes in your management of patients with diabetes in your practice in the past two years?
2. What in your opinion were the factors that led to change your management of patients with diabetes in your practice in the past one to two years?
3. In your opinion, how did the TARGET meeting on diabetes affect how you managed patients with diabetes in your practice?
4. Did you make any changes as a direct result of the TARGET meeting?
5. What were these changes?
6. If it did lead to change, what features of the TARGET meeting do you believe enabled this change?
7. What other factors do you believe (may have) led to change in your practice?
8. What do you feel were possible barriers to this change and how important were they in preventing change?
9. How, if at all, did you seek to overcome these barriers?
10. How have you managed to sustain change?

Thematic analysis was used to make sense of the data. This involved examining the transcribed interviews to identify key issues and then coding and categorising text expressing these recurrent issues to form explanatory themes.

#### Ethics committee

Lincolnshire Research Ethics Committee (study number: 02/1/680). The study was approved for research management and governance by West Lincolnshire PCT.

## Results

101 practices, 38 from East Lincolnshire, 25 from Lincolnshire South, and 38 from West Lincolnshire were included in the study. Of these, 15 were training practices, 56 Personal Medical Services (PMS), 18 single-handed practices and 59 dispensing. At least one GP attended the intervention session from 68 practices, and from 64 at least one practice nurse or health visitor attended. No clinical professional attended from 25 of the practices. From 12 practices only one or more GPs attended and no nurses, from 8 practices one or more practice nurses or health visitors but no GPs, and from 56 at least one GP and at least one practice nurse or health visitor attended. All 101 practices had their prescribing data analysed for ACE inhibitor prescribing before and after the educational session and the quantitative analysis has been described in detail in a separate paper [10].

The results have been written under the five main themes emerging from the data which include changes in processes of care, facilitators for change, barriers to change, sustaining change and perceived effect of PLT (See Table 3). In order to differentiate between the different strata and also the professional group of the interviewee, each quote was identified as such, for example stratum one, GP quote (S1 GP), or stratum three nurse (S3 NS). The effect of stratum on change was also explored.

**Table 3:** Themes and subthemes

**Changes in processes of care**
*Protocol development*
*Policy review*
*Changes in clinical management*

**Facilitators for change**
*Evidence and guidance*
*Information sources*
*Clinical audit*
*Information technology and decision support*
*Role redesign*
*Training in diabetes*
*Patient involvement and empowerment*

**Barriers to change**
*Inertia*
*Lack of resources*
*Risks versus benefits of medication*
*Lack of secondary care support*

**Sustaining change**
*Systematic attention to detail*
*Team approach/holistic care*
*Personal interest and enthusiasm*

**Perceived effect of TARGET on diabetes care**
*Specific changes made as result of TARGET attendance*
*Peer pressure in TARGET*
*Lack of change from TARGET*
*Use of consultants in TARGET*

### Changes in processes of care

The main changes stated by interviewees in the care of patients with diabetes in the year following the educational intervention included increased used of protocols and policies; using ACE inhibitors as a first line for patients with diabetes who developed hypertension; switching patients to glitazones; increased use of aspirin and statins; putting patients on insulin either directly or by referral to secondary care; quicker titration of drugs leading to increased prescriptions; increased screening and review of patients with diabetes. Practices in strata 1 and 2, though still aspiring to improve care were less able to specify the precise interventions by which they did this.

### Protocol development

"...We've had a diabetes protocol in existence for about 4 or 5 years...it's now in its third different edition...because we did the 'Micro-Hope' and NSF as a single editing and its now been redone for the Quality and Outcomes Framework..." (S4 GP)

### Policy review

"Well we've reviewed the policy of putting all the hypertensive patients on ACE inhibitors ... and ACE inhibitors as a first line for all diabetic patients who turned hypertensive after they developed diabetes." (S4 GP)

Increased review leading to additional prescribing "We're seeing diabetic patients much more regularly than we did, and we're noticing changes in their blood pressure and cholesterol levels, making sure they have more screenings and all their checks are in place." (S1 GP)

### Changes in clinical management

"...We are using more glitazones than we were and having some very good results..." (S3 NS)

"... [We] put most of the diabetic patients on aspirin, the majority of them on a statin regardless of their cholesterol level." (S3 NS)

"...We just referred the tenth patient to the nurse [for insulin], those ten patients otherwise would have been referred to hospital." (S4 GP)

"We've picked up a lot of new diabetics, simply by the recall system we have for our CHD patients and a lot of them are turning up..."(S1 GP)

"...If you come in for a blood test you tend to get a glucose done and it ifs high we then take it further we don't just think its slightly high, you know, watch your diet, we do tend to do a diagnostic for a blood glucose test." (S3 NS)

### Facilitators for change

There were a number of explanatory factors offered by participants as leading to these changes in diabetic care. These ranged from dissatisfaction with current services to government initiatives based on the outcomes of major research studies. The main reported facilitators of change were financially driven performance targets, particularly those set by the new GP contract and quality and outcomes framework, large research studies such as HOPE/Micro HOPE, and UKPDS as well as government guidelines as outlined in the NICE guidance and Diabetes NSF.

### Incentives and resources

"We can now use the stream of resources coming in that are tied to quality, to employ people and health care assistants" (S4 GP)

"What's happened with the new contract is that you have been given a financial incentive to do it, to record it and to make it available for auditing and for PCTs to make sure you have done it..." (S2 GP)

### Evidence and guidance

"We decided as a practice after the publication of 'HOPE' that we would take an opportunistic approach to starting diabetic patients on ramipril when we saw them and that's been reasonably successful in bringing our rates of ramipril prescription up.... "(S4 GP)

"UKPDS was such a huge trial...so you couldn't miss it unless you were blind...it's not that they were telling you something new but it does have an input." (S1 GP)

"And of course all the NICE guidance that came out, particularly with the use of diabetes zones, the drug therapies, foot care guidance that was out in January, that's influenced the way we refer to podiatry and how the podiatry work with us now." (S3 NS)

"Evidence from the National Service Framework...NICE and clinical guidelines... so probably all those things, the most important would probably be the NSF I would think." (S2 GP)

### Information sources

Information was received from a number of sources including the Primary Care Organisations and professional journals but drug representatives were also seen as an important source of information.

"The National Service Framework regarding diabetes and protocols that have come in from the Trust." (S2 GP)

"Well I take a couple of these diabetes and CHD combined journals...and then if it's of particular interest to me or particularly pertinent to a patient then I might look it up." (S3 NS)

"I think they [drug representatives] probably played a bit more of a part here that they usually let them do which is that they did persuade me that glitazones were probably a good thing..." (S4 GP)

### Clinical audit

Audit and benchmarking care against other practices' performance was perceived to be an important driver for change in all practices interviewed. Clinicians in most practices used 'real-time' entry to improve data recording but for some practices this was a relatively recent phenomenon.

"...We've taken a sample of about 50–60 patients we've picked up at random and we've followed them right the way through and it's a report back but a lot of things have changed over those two years so its quite interesting." (S3 NS)

"You're in line with your colleagues and thinking along the same line I think sort of group approach... everybody's calling the same tune, at least within this county." (S1 GP)

### Information technology and decision support

"We're all using the computer live and we've being doing so for quite a while, it's take a bit of a churn to get people to do it but it's working." (S1 GP)

"We've got a very slight detailed template...we've got extra things on that we like and we also have sub templates for podiatry and eye screening retinopathy..." (S3 NS)

### Role redesign

A number of practices encouraged nurses, particularly the diabetes nurse, to take a leading role in delivery. Health care assistants also supported diabetes care, either working alongside the nurse or to protocols supervised by the general practitioner (GP). GPs appreciated that using health care assistants in this way was a radical change.

" [We are] encouraging the nurse practitioner to take on more of a leading role..." (S1 GP)

"Our diabetic nurse...we do tap into her, her sources and her skills, and if we have something difficult we will turn to her rather than a hospital clinic...""the health care assistants do the blood tests,...height,...weight, the blood pressure, they check that they're seeing the chiropodist, ... the opticians and they fill in as much as they can." (S3 GP)

### Training in diabetes

Training of professionals in the care of diabetes was also seen as a facilitator of change. By contrast one practice in stratum 1 felt that he was under too much other pressure to consider training in diabetes.

"I think everybody who sees a diabetic patient should have had some training, you know, not just the minimum, hide behind the one day, diabetes management course its got to be something much more encompassing the complexities of the disease." (S3 NS)

"I went to the diabetes UK conference two years ago and came back from that really fired up, so the doctors have very much let it be led by me..." (S3 SN)

### Patient involvement and empowerment

Clinicians interviewed expressed ambivalence about patient empowerment. Some clinicians felt that patients were increasingly knowledgeable about their condition, often using the Internet for information, and this affected how they provided services.

Giving patients written information about their condition was seen as empowering. Some practices offered routine annual reviews whilst others actively encouraged patients to decide when to contact the practice for additional care or advice. Patient held records were also mentioned by one practice that felt that they helped inform patients about what had been done for them.

"I was looking at ways in which to empower the patients and what that empowerment actually meant to people. What we actually discovered was, empowerment meant different things to different people." (S1 GP)

"A lot of patients would really we 'did' it for them.... I'm afraid I'm a bit sceptical about patient empowerment, I think there are patients who do but I'm not sure that it's as quite as wide spread...as the enthusiasts for it would have you believe." (S4 GP)

"What we do is send off a pack with various nutrition information...that sort of stuff, together with their annual blood forms and things like that so that when they come back we then invite them back to the clinic." (S1 GP)

"Because we only recall our patients once a year with having so many we rely on them doing the blood test and coming in if there's a change and telling us, I mean that again is patient education but we spend a lot time sort of explaining that if there's a big change...don't wait..."(S3 GP)

### Barriers to change

Inertia was cited as a hurdle to change. One of these practices had undergone significant change already, the other less so. Other stated barriers included resources, concerns over doing more harm than good with medication and lack of secondary care support.

### Inertia

"I think that partly it's the inertia in the system that when you're doing things and the results show you that you're doing pretty well there's a tendency to say, well fine, don't need to put much more effort into changing things here..." (S4 GP)

"The most difficult thing is to then make the change... going back and deciding how we going to get better, making sure everyone knows about it and discussing it and finding the time to go over it. I think we've done okay." (S2 GP)

### Lack of resources

"I work twenty-six hours a week and I could spend every hour of that doing diabetics, I don't because I'm a nurse practitioner as well and with us being short-staffed at the moment..." (S3 NS)

"Time ... diabetes is not the only thing to get done, so it is the practice staff time." (S1 GP)

"Financial, lots of space and more opportunity for education." (S1 GP)

"Yes, there's two big barriers, one is time and one is money and that's the two big things. I mean if we could fund more hours for diabetic patients [although] we couldn't at the moment because of the building space..." (S3 NS)

### Risks versus benefits of medication

One GP felt concern about putting patients who appeared otherwise healthy onto an increasing number of potentially harmful medications in order to meet targets.

"I've got somebody in their late seventies...who's had type 2 diabetes for decades I find it very difficult motivating myself to get her cholesterol down. I find that very difficult to defend, and I appreciate that somebody else might say that you shouldn't be allowed to do that... Occasionally I think why am I making this woman potentially ill with medication...blood pressure reduction can be achieved ultimately but it may take four or five different approaches per patient and we have to ask ourselves, is that in the patient's best interest and all these drugs?" (S2 GP)

### Lack of secondary care support

"Dietician support ... at the moment it is pie in the sky, I think its taking months and in fact the dietician have recently written to us suggesting that because they're snowed under, we get our practice nurses trained in what needs to be done for diabetic assessment.... There's always more demand than supply." (S2 GP)

### Sustaining change

Key factors in sustaining change were a systematic and team based approach with enthusiastic clinicians who had a personal interest in diabetes care.

### Systematic attention to detail

"...getting into the habit so that when you see a patient coming back for review you try and make it automatic, should they be on an ACE inhibitor, do they need to be on a statin, is their HBA1C still coming down or at least stable, so its really a question of getting into habits of it which is why a diabetic clinic is so advantageous because diabetes care more than anything else I think is a matter of attention to detail." (S4 GP)

### Team approach/holistic care

"I think it's the way you go about the change in the first place...what we tend to do is take an idea to the team and let them thrash it out and find out what the answers going to be." (S3 NS)

"...by combining diabetes and care of its complications particularly Coronary Heart Disease together we actually care for patients fairly holistically, they don't on the whole go to the diabetic clinic and the Coronary Heart Disease clinic, we found that we do them all in one go if we possibly can so that's time saving..."(S4 GP)

### Personal interest and enthusiasm

"It's the enthusiasm with which I carry something through that becomes infectious and that's why the change, it's not all down to me, it's like a domino effect." (S3 NS)

"I mean obviously it's been driven partially by the fact I have an interest in diabetes anyway..." (S1 GP)

### Perceived effect of TARGET on diabetes care

Participants expressed that the research outcomes of major studies and government policy had a big impact on practices. What difference did the protected learning time session for diabetes make? Only one practice interviewed stated that they had not attended TARGET. This GP was a single-handed practitioner in stratum 1 and gave 'lack of time' as his reason for non-attendance. Practices were mixed in their opinions about whether TARGET had made a difference in their care provision for patients with diabetes. Although many felt that they were already implementing change the majority of practices cited new systems such as screening at-risk patients, providing information for receptionists, organising foot care and putting patients on aspirin as resulting directly from TARGET.

Group work was specifically mentioned as helpful by two practices where working with 'fellow GPs', colleagues from different disciplines and other practices was seen as beneficial. Peer pressure and being seen to be 'in line' with colleagues appeared to be another important factor. In-house training using protected time was mentioned by two practices in strata 3 and 4 as a way of developing their individual practice's systems. Expert speakers (non GP) had a limited appeal to some practitioners whereas other general practitioners were welcomed.

### Specific changes made as result of TARGET attendance

"Yes, that came from TARGET when we did the screening for Diabetes, I remember being in the discussion about what's the most economical way to detect the patients with undiagnosed diabetes and as a consequence of that we target all our chronic disease people for screening for diabetes..."(S3 NS)

"...the girls from the reception found it particularly useful meeting people with diabetes and discussing the ins and outs, they haven't been quite so aware of Hypos and blood tests and things like that..."(S3 NS)

" [I] did go along to the TARGET morning that they had on foot care in diabetes and that did modify my approach to examine diabetic feet which has had a knock-on effect, we're pretty confident about doing them so that they're now pulling out podiatry for examining diabetic feet apart from more complicated ones..."(S4 GP)

"Aspirin was one of the things that was brought up from the TARGET meeting because we've been discussing it already whether to put diabetics on aspirin and they did discuss the HOPE study that really pushed along to decide that yes, we were going to put people on aspirin, so yes that came directly out of TARGET..." (S3 NS)

### Peer pressure in TARGET

"I did feel that there was like quite a big gap between people who were doing their very best and the people who were not doing very well at all." (S3 NS)

"If you're thinking of being more pro-active putting someone on an inhibitor and you go along and every other GP says "Oh yeah, that's a good idea, we're doing that", it will mean that you do it, you get peer pressure to do it. If it was just one doctor with a specialist interest [saying] by the way the latest research shows this and all the GPs say 'yeah we'll do that next time." (S2 GP)

### Lack of change from TARGET

"It didn't make any difference here because we were already doing most of what was done, ... the actual care in the practice was already beyond what TARGET was aiming for anyway". (S3 NS)

"No, because I didn't go to the meeting...I'm a single handed practitioner and it's not as though I've got to travel a million miles to target, but it's the time factor..."(S1 GP)

### Use of consultants in TARGET

"...It's nice to see the consultant from time to time but it's best practice in the situations that we find ourselves in and I think there's a tendency to get a little tired with the experts telling us..." (S4 GP)

### Effect of stratum on change

Practices in strata 1 and 2 (little or some change before and after the educational intervention) generally reported making opportunistic rather than systematic improvements whereas strata 3 and 4 practices (a great deal of change before or after the educational intervention) tended to cite influencing factors and resulting changes in practice more often. Changes in processes were report to occur in every practice to some extent, but for participants from strata 3 and 4 described change at a faster pace. Practices in stratum 4 in particular tended to have been early adopters, one practice in this group stating that they started implementing 'HOPE' very soon after publication. Practices in strata 3 & 4 were more likely to mention utilising protected learning time for in-house training in order to develop the practices' systems and teamworking. Practices in strata 3 & 4 were also more likely to acknowledge the benefit of diabetes education whereas those practices in stratum 1 reported themselves to be under too much pressure from other areas of work to undergo additional education.

## Discussion

### Principal findings

Despite some interviewees reporting that protected learning time (PLT) had not been a major influence in bringing about change in prescribing, others indicated that PLT was one of a number of key drivers for bringing about change in diabetes care in their practice [11]. Changes occurring after the educational intervention included using ACE inhibitors as a first line for patients with diabetes who developed hypertension, increased use of aspirin, switching patients to glitazones and commencing insulin; such changes by leading to better control of glucose and cardiovascular risk factors are known to reduce complications of diabetes.

Reported facilitators of change were financially driven performance targets, research evidence and national guidance. Despite many interviewees suggesting that they had already looked at diabetes care provision and that the educational session may not necessarily have influenced this, most practices offered explicit examples of change introduced directly as a result of the session. Other factors for change linked to the educational session were peer support, teamworking and benchmarking through audit and comparative feedback.

### Strengths and weaknesses

The main strengths of the study were that it provided an explanatory framework for changes linked to a quantitative study into the effect of protected learning time on prescribing, that the practices were stratified for inclusion according to change in prescribing and that prescribing status was blinded by both interviewee and interviewer. Limitations included the small number of practices from each stratum and the potential for recall bias. Respondent validation, or other data validation, was not undertaken.

### Context of other literature

The findings support the role of peer influence and modelling in the learning process [12] which was more than simply from networking opportunities [2]. The protected learning session provided influential sources of information and delivered a personalised message, based on individual experience focusing on specific evidence linked to clear outcomes and encouraging change [13]. Local opinion leaders and early adopters who contributed to the educational programme may have had a beneficial effect on adoption by others [14] and the interactive nature of the educational process was more likely to improve outcomes compared to didactic lectures [15], a view supported by adult educational theory [16]. Respondents highlighted audit and benchmarking as facilitators for change and although evidence for this from the literature is equivocal [17] it could be argued that the audit process was a mechanism for peer influence as well as providing a basis for measurement of change. Interprofessional learning may also have had a positive impact [8] given that members of practices teams had the opportunity to discuss potential changes during the session. This approach was similar in some respects to the academic detailing approach for practice teams [18].

## Conclusion

This study has shown how a protected learning time scheme, using local opinion leaders and early implementers as change agents and audit and feedback, was one of a number of factors supporting changing systems of diabetes care in some practices. Utilising a combination of approaches to address barriers to change [19] was integral to the concept of the protected learning time scheme. The educational session addressed barriers to change, known to be helpful in modifying outcomes, by sharing learning across practices [20]. Various other evidence based strategies to improve performance, such as identifying with the concerns of practitioners and patients, using practice-based active learning methods, delivery by opinion leaders and peers, encouraging collaboration and teamwork were employed as part of the teaching programme [21].

## Competing interests

The author(s) declare that they have no competing interests.

## Authors' contributions

ANS had the initial idea, contributed to the design and led the study. JM and CW conducted the interviews. JM and KS undertook the data analysis. All authors read and approved the final version of the paper. ANS is the guarantor for the paper.

## Pre-publication history

The pre-publication history for this paper can be accessed here:


